# Highly Processed Food and Functional Gastrointestinal Disorders in Children and Adolescents with Obesity: The Preventive Challenge—A Narrative Review

**DOI:** 10.3390/nu17233744

**Published:** 2025-11-28

**Authors:** Valeria Calcaterra, Hellas Cena, Chiara Ferrara, Vittoria Carlotta Magenes, Sara Boussetta, Ilaria Zambon, Gianvincenzo Zuccotti

**Affiliations:** 1 Department of Internal Medicine and Therapeutics, University of Pavia, 27100 Pavia, Italy; valeria.calcaterra@unipv.it; 2 Pediatric Department, Buzzi Children’s Hospital, 20154 Milan, Italy; vittoria.magenes@unimi.it (V.C.M.); gianvincenzo.zuccotti@unimi.it (G.Z.); 3 Laboratory of Dietetics and Clinical Nutrition, Department of Public Health, Experimental and Forensic Medicine, University of Pavia, 27100 Pavia, Italysara.boussetta01@universitadipavia.it (S.B.); ilaria.zambon01@universitadipavia.it (I.Z.); 4 Clinical Nutrition Unit, Maugeri Scientific Clinical Institutes—Scientific Institute for Research, Hospitalization and Healthcare, 27100 Pavia, Italy; 5 Department of Biomedical and Clinical Science, University of Milano, 20157 Milan, Italy

**Keywords:** high processed food, functional gastrointestinal disorders, children, adolescents, obesity, prevention

## Abstract

**Objective:** This narrative review summarizes current evidence on the associations between ultra-processed food (UPF) consumption, childhood and adolescent obesity, and functional gastrointestinal disorders (FGIDs), and examines the metabolic, inflammatory, microbial, and gut–brain mechanisms underlying these links. **Methods:** A comprehensive search of PubMed and Scopus identified articles published between January 2010 and September 2025. Eligible studies included human research in individuals aged 0–18 years; adult studies were considered when contributing relevant mechanistic insights. Of 335 records screened, 112 studies met the inclusion criteria and were synthesized narratively according to methodological appropriateness. **Results:** High UPF intake was consistently associated with increased adiposity, metabolic dysregulation, and greater cardiometabolic risk in youth. Children with overweight or obesity showed a higher prevalence of FGIDs compared with their normal-weight peers. Mechanistic evidence suggests that UPFs, rich in refined carbohydrates, unhealthy fats, and additives, may promote gut microbiota dysbiosis, impair intestinal barrier integrity, alter motility, and induce low-grade inflammation, thereby disrupting gut–brain signaling and contributing to FGID symptoms. Early-life and maternal UPF exposure may further increase susceptibility to metabolic and gastrointestinal disturbances through epigenetic and microbiome-mediated pathways. **Conclusions:** UPFs emerge as a shared etiological factor for obesity and FGIDs in childhood. This review contributes an integrated synthesis of epidemiological and mechanistic data while highlighting key research gaps, particularly the need for standardized methodologies and pediatric interventional studies to strengthen the evidence base.

## 1. Introduction

Childhood obesity has become a major global public health concern, with over 160 million school-aged children currently affected [1]. Although the development of obesity arises from a multifaceted interplay of genetic, epigenetic, environmental, and behavioral influences, lifestyle remains the pivotal element capable of modulating this risk. In particular, dietary patterns play a decisive role, as they represent both a major determinant and a potential target for prevention. Among lifestyle factors, dietary patterns play a decisive role, as they represent both a major determinant and a potential target for prevention. In this context, the widespread intake of ultra-processed foods (UPFs), industrial formulations composed mainly of refined ingredients such as oils, sugars, starches, and proteins, and containing little or no whole foods and often enriched with additives to enhance palatability, texture, and shelf life [2], has gained increasing attention as a central factor contributing to the deterioration of diet quality in children and adolescents. A substantial body of evidence links high UPF consumption to greater adiposity and metabolic disturbances in youth: a 2024 systematic review by Petridi et al. [3] reported that the vast majority of studies found a positive relationship between UPF intake and overweight or obesity in children and adolescents, while frequent consumption was associated with increased waist circumference, dyslipidemia, and insulin resistance even at early ages. Similarly, Mescoloto et al. [4] highlighted that UPFs now provide a substantial proportion of total energy intake among youth globally, with such dietary patterns systematically associated with excess weight and elevated cardiometabolic risk.

Beyond caloric surplus, these foods pose an additional challenge due to their hyper-palatable and rewarding characteristics. Engineered to stimulate pleasure and cravings, UPFs may activate brain reward pathways similar to those implicated in addictive behaviors [5]. Regular exposure during childhood can condition strong preferences for sweet, salty, and fatty flavors, diminishing appetite for nutrient-dense foods such as fruits and vegetables [2]. Consequently, reward-driven eating behaviors reinforce overeating and weight gain [5], while habits like frequent snacking and meal skipping, often involving UPFs, are associated with higher obesity risk.

Although obesity is commonly linked to cardiometabolic disorders such as type 2 diabetes, hypertension, and fatty liver disease [1], growing evidence suggests that it may also predispose to functional gastrointestinal disorders (FGIDs).

FGIDs, recently termed disorders of gut–brain interaction, are chronic gastrointestinal conditions characterized by recurrent symptoms such as abdominal pain, bloating, and altered bowel habits in the absence of identifiable structural abnormalities [6,7]. Pediatric FGIDs, including irritable bowel syndrome, functional dyspepsia, abdominal pain disorders, and constipation, represent a leading cause of referral to pediatric gastroenterology services [6,8]. Multiple studies indicate a higher prevalence of FGIDs among children with overweight or obesity compared to their normal-weight peers [9,10,11]. Obesity is associated with low-grade systemic inflammation, which can sensitize visceral afferents and amplify pain perception; altered gastrointestinal motility, predisposing to symptoms such as constipation or postprandial distress; and gut microbiota dysbiosis, driven in part by diets rich in refined carbohydrates and UPFs [11,12,13,14,15,16,17,18]. Additionally, obesity-related non-alcoholic fatty liver disease may interfere with bile acid metabolism, further influencing motility and visceral sensitivity. Psychosocial stressors, more prevalent in children with obesity, may also exacerbate FGID symptoms via dysregulation of the gut–brain axis.

In light of these findings, dietary patterns dominated by UPFs may represent a shared etiological link between obesity and FGIDs. Diets rich in refined carbohydrates, unhealthy fats, and food additives but poor in fiber can disrupt the gut microbiome, impair intestinal barrier integrity, and alter motility and gut–brain signaling—all of which may exacerbate FGID symptoms [19,20,21,22].

This narrative review aims to synthesize current evidence on the relationship between UPFs consumption, childhood obesity, and FGIDs. Specifically, it seeks to elucidate the potential mechanisms, metabolic, microbial, inflammatory, and neuro-gastrointestinal, through which UPFs may contribute to both excess adiposity and FGID pathophysiology, and to identify gaps in existing research that can inform prevention and clinical management strategies.

## 2. Methods

This narrative review was conducted with the objective of synthesizing and critically evaluating the available evidence on the associations between UPF consumption, childhood and adolescent obesity, and FGIDs, as well as exploring potential mechanisms involving the gut–brain axis. The review followed a structured but flexible narrative approach, aimed at integrating findings from epidemiological studies, clinical trials, and mechanistic research.

### 2.1. Search Strategy

A comprehensive literature search was performed using two electronic databases—PubMed and Scopus. The search covered publications from January 2010 to September 2025 and was restricted to studies written in English. The population of interest included human subjects aged 0–18 years; however, studies conducted in adult populations were also included when they contributed valuable mechanistic insights relevant to pediatric conditions.

The search strategy combined predefined keywords and Boolean operators. Key terms included: “childhood obesity,” “adolescent obesity,” “ultra-processed foods,” “functional gastrointestinal disorders,” “gut–brain axis,” “microbiota,” “diet,” “stress,” and “psychology.” The search strings were adapted for each database to maximize sensitivity and accuracy.

Eligible records included original research articles (observational or interventional), as well as narrative or systematic reviews that provided relevant conceptual or mechanistic information. Exclusion criteria comprised: animal or in vitro studies; non-English publications; papers not directly addressing UPFs, obesity, FGIDs, or related pathophysiological mechanisms.

### 2.2. Study Selection Process

The initial database search identified 335 records. Titles and abstracts were screened independently by two reviewers to assess relevance and identify duplicates, leading to the exclusion of 173 articles. The remaining 173 full-text articles were then examined in detail against the eligibility criteria. Ultimately, 112 studies met the inclusion criteria (65 focusing on pediatric populations and 47 including mixed-age populations). To ensure completeness, manual searches of reference lists from the included papers were performed to capture additional relevant studies.

### 2.3. Data Extraction and Synthesis

For each included article, key information was extracted regarding study design, population characteristics, exposure definitions, outcomes, and main findings. Given the heterogeneity of study types and methods, a narrative synthesis approach was adopted, allowing the integration of quantitative and qualitative evidence. Findings were grouped thematically to address the review objectives: the relationship between UPF intake and obesity in children and adolescents; the association between UPFs and FGIDs, and potential biological and psychosocial mechanisms linking diet, obesity, and gut–brain axis alterations.

## 3. Results

### 3.1. Ultra-Processed Foods

UPFs are industrially manufactured products that mainly consist of substances extracted from foods, such as fats, oils, free sugars, starches, and amino acids, or are derived from food components. These foods contain little or no whole ingredients and are often mixed with additives, such as colorants, stabilizers, humectants, and emulsifiers, which are not typically found in home kitchens. UPFs are designed to be convenient and highly palatable, and they have a long shelf life due to the manufacturing processes they undergo [23].

#### 3.1.1. UPF Classification Systems

There are several systems used to classify foods based on their level of industrial processing. The NOVA classification system is the most widely recognized and internationally accepted method for categorizing foods according to their level of industrial processing. Foods are divided into four main groups: unprocessed or minimally processed foods; processed culinary ingredients; processed foods; and UPF [24].

In addition to NOVA, there are other classification methods, such as the EPIC (European Prospective Investigation into Cancer and Nutrition) system and the Siga classification, which are used less frequently. Unlike the NOVA system, which offers a broad definition of UPFs, the EPIC approach provides specific definitions of processing levels for each food category. It divides foods into three main groups: highly processed, moderately processed, and unprocessed [25]. The SIGA classification, on the other hand, enhances the NOVA system by incorporating its four groups along with additional subcategories that consider factors such as the effects of processing on the food or ingredient matrix, the amounts of added salt, sugar, and fat, the number and types of ultra-processing indicators, and the presence of potentially harmful additives [26].

#### 3.1.2. Dietary Patterns and Nutrient Deficiency Issues

Regardless of the classification system used, the link between the excessive consumption of UPFs and negative health implications is undeniable. The consumption of UPFs in Italy is lower than in non-Mediterranean countries, such as the United States (US) and the United Kingdom (UK) [27]. However, data from the childhood obesity surveillance program (Okkio alla Salute), indicates that UPFs still constitute a significant and growing portion of the diet, accounting for 36% of total energy consumption, especially among the youngest age group (8 to 9 years) (Epicentro-ISS Istituto Superiore di Sanità Sistema Di Sorveglianza Okkio Alla SALUTE 2019) [28]. These data are also confirmed by a further examination of the Italian Nutrition & Health Survey (INHES) cohort [27], which includes children and adolescents aged between 5 and 19 and is conducted between 2010 and 2013. On average, UPFs account for 25.9% of total energy intake among children and adolescents, almost double the share observed in Italian adults (17.3%). The main categories of UPFs contributing to this energy intake, in order of importance, are processed meats, sweet biscuits, cakes, croissants, and other non-artisan pastries, as well as sugary drinks. Other relevant categories, though contributing less, include fruit yogurt (8.9%), sliced cheese (7.2%), breakfast cereals and bars (6.3%), packaged bread, industrial pizza, ready meals, and packaged sweet and savory snacks [27].

Consumption patterns of UPFs show significant differences between industrialized and developing countries, despite some similarities. In high-income countries (HICs), UPFs account for a substantial portion of energy intake, often exceeding 50–60% of daily calories, with particularly high consumption rates among children and adolescents. Common contributors to UPF intake in this demographic include baked goods, packaged snacks, and processed cereals [29,30]. On the other hand, low- and middle-income countries (LMICs) typically report lower UPF consumption, usually between 18% and 35%. However, these countries are experiencing the fastest growth rates in UPF consumption, especially in urban areas and among higher socioeconomic groups. In LMICs like Brazil, India, Ethiopia, Uganda, and Lebanon, diets are increasingly influenced by sweetened beverages, biscuits, and sweetened dairy products. Alarmingly, there is evidence of these foods being introduced at an early age during infancy [30,31,32,33,34].

A diet high in UPF tends to exhibit a reduced variety of food sources, which is linked to lower dietary quality. This decrease in quality increases the risk of developing noncommunicable diseases and nutritional deficiencies in adults [35]. Since children and adolescents consume more UPF, they may face an even greater risk. Research from the United States, Brazil, and Taiwan shows that diets rich in UPFs are strongly associated with nutrient deficiencies, particularly in calcium, magnesium, phosphorus, zinc, chromium, iron, selenium, and vitamins A, C, D, E, niacin, and pyridoxine. These deficiencies can compromise children’s growth and development [36]. The consumption of these foods most frequently occurs at specific times of the day, such as during breakfast, while watching TV, or when using computer [37]. Higher consumption of UPFs is linked to a diet that moves away from the principles of the Mediterranean diet, and is characterized by higher sugar and sodium intake and lower total carbohydrate and fiber intake [27]. In fact, UPFs are a primary source of free and added sugars in the modern diets of children and adolescents. Studies conducted in various countries show a direct, dose–response relationship between the proportion of UPFs in the diet and the amount of free and added sugars consumed, which account for almost 70% of the total sugar intake from these products [30]. Added sugars are commonly found under names such as high-fructose corn syrup, invert sugar, maltodextrin, and dextrose, ingredients that are rarely used in home cooking. Consuming large amounts of UPFs complicates adherence to the World Health Organization’s recommendations about the added sugar (<10% of the total energy intake), increasing the risk of chronic diseases such as diabetes, childhood obesity, and other metabolic disorders [38,39].

Additionally, the soft texture of these foods often reduces chewing time, which can delay the signals that indicate fullness, leading to excessive consumption [40]. A randomized clinical trial conducted by Chen et al. (2020) finds that a diet high in UPF results in participants consuming approximately 500 extra calories per day, leading to weight gain compared to a diet based on unprocessed foods [30].

Diets that are high in UPFs typically contain low levels of healthy fats, such as omega-3 polyunsaturated fatty acids, while being high in monounsaturated fatty acids. Trans fats, which are industrially produced and derived from partially hydrogenated oils, are often added to these products to extend their shelf life and enhance their texture. An increased consumption of UPFs is linked to a higher intake of saturated fats, which in turn raises the risk of developing cardiovascular disease [41].

Although UPF consumption is still lower in Mediterranean countries compared to other regions, it is steadily increasing and has already become a substantial part of the diet among Italian youth. This elevated consumption of ultra-processed foods reduces overall diet quality and is associated with a higher risk of nutritional deficiencies and chronic diseases, highlighting the need to limit their intake and promote healthier, more sustainable dietary patterns.

### 3.2. Pediatric Obesity and Functional Gastrointestinal Disorders

#### 3.2.1. Epidemiology of Pediatric Obesity

Childhood obesity has become a critical public health issue worldwide, with prevalence rates rising dramatically in recent decades. Globally, the proportion of youth classified as having overweight or obesity (ages 5–19) increased from just 8% in 1990 to approximately 20% by 2022 [42,43]. This surge translates to over 390 million children and adolescents above a healthy weight in 2022, including about 160 million with obesity [42,43]. The epidemic spans high-income and low-income regions alike: while some high-income countries saw obesity rates plateau in the late 2010s, many low- and middle-income countries have continued to experience rising childhood obesity rates [44]. Notably, the COVID-19 pandemic appears to have exacerbated the trend, as lockdowns and lifestyle disruptions were associated with accelerated weight gain among children in several regions [44]. In the United States, recent estimates indicate that roughly 1 in 5 adolescents (about 21%) meet the criteria for obesity [45]. Similar patterns are observed in Europe and other parts of the world, signaling that pediatric obesity is a widespread challenge with no signs of abating [46].

Importantly, obesity in youth is linked not only to future cardiometabolic diseases but also to a broad spectrum of psychosocial (e.g., stigma, anxiety, depression) and physical complications and reduced quality of life even in childhood [44,47,48]. Concerning physical comorbidities, excess adiposity in childhood confers a range of direct and indirect gastrointestinal consequences [49,50,51,52].

#### 3.2.2. Pediatric FGIDs: Definitions and Prevalence

Less appreciated, but increasingly recognized, is the association between obesity and FGIDs [7]. FGIDs, recently re-termed disorders of gut–brain interaction, are a group of chronic GI conditions characterized by symptoms (pain, bloating, altered bowel habits, etc.) in the absence of identifiable structural abnormalities [6]. Common pediatric FGIDs include irritable bowel syndrome (IBS), functional dyspepsia, functional abdominal pain, and functional constipation (FC) [1]. These disorders are common in the general pediatric population, affecting roughly 1 in 5 children according to global estimates [6], and are a leading cause of pediatric gastroenterology visits [8]. Traditionally, FGIDs have been linked to disordered motility and visceral hypersensitivity modulated by the gut–brain axis, with triggers including stress and certain foods [53]. Now, emerging evidence suggests that obesity is another risk factor for FGIDs in children [1,54].

Numerous pediatric investigations have reported that FGIDs are more frequent in children and adolescents with overweight or obesity than in their normal-weight counterparts [9,10,11] and that obesity is associated with poor outcome and disability at long-term follow-up in children with abdominal pain-related FGIDs [55].

#### 3.2.3. Association Between Obesity and FGIDs

Several studies support a strong relationship between excess weight and FGIDs in childhood (Table 1). In a pediatric gastroenterology referral population, Teitelbaum et al. [54] found that in a pediatric gastroenterology referral population, 23% of children with FC and 24.8% of those with IBS had obesity, rates significantly higher than in the control group. Another study by Galai et al. [10] showed that 39.5% of adolescents with functional abdominal pain (FAP) were overweight or obese, versus 30% in healthy controls [11,56]. Similarly, Tambucci et al. [11] noted FGIDs to be markedly more common in youths with excess weight (47.6%) than in normal-weight children (17.4%). Interestingly, the authors showed that FC, functional dyspepsia, and IBS occurred significantly more often in the overweight/obesity subjects, whereas FAP rates did not differ appreciably by weight status. Consistently, Phatak et al. [57] reported a higher overall prevalence of FGIDs in children with overweight/obesity (16.1%) compared to their normal-weight counterparts (6.9%). In their work, nearly half of the children with excess weight had at least one FGID, underscoring the frequent coexistence of these conditions [57]. Notably, a review by Zia JK et al. [58] concluded that, overall, there is a clear link between excess weight and functional GI disorders in childhood, even if differences emerge by subtype (with obesity’s link to constipation being most consistently observed). These patterns mirror data in adults: a 2012 meta-analysis by Eslick et al. [59] had likewise shown that obesity is significantly associated with various GI symptoms (especially upper-abdominal symptoms like reflux and bloating) in adult populations. Among the various pediatric FGIDs, the strength of association with obesity appears to differ by subtype. Indeed, the link between excess weight and functional constipation is particularly important [56,60]: a large retrospective study of 719 children with chronic constipation found obesity in 22.4% of cases, compared to 11.7% in a control pediatric population [57]. This disparity was especially pronounced in male patients [57]. By contrast, findings for conditions like IBS have been more inconsistent across studies, with some pediatric cohorts showing only modest or non-significant weight-related differences [56]. This heterogeneity in results, such as the stronger obesity link observed in constipation relative to IBS, may stem from underlying differences in gut motility, dietary patterns, or hormonal influences for each disorder [56]. Furthermore, Bonilla et al., in a prospective cohort study in an outpatient clinic-based sample of patients diagnosed with abdominal pain-related FGIDs, noted that obese patients were more likely to have abdominal pain, with higher intensity and higher frequency, than non-obese patients. Moreover, obese children with FGIDS had more school absenteeism and disruption of daily activities at follow-up than normal weight patients [55].

#### 3.2.4. Mechanisms Linking Obesity to FGIDs

The relationship between obesity and FGIDs in children results from a complex interplay of dietary, microbial, inflammatory, psychological, and mechanical factors. Unhealthy dietary patterns typical of pediatric obesity are characterized by high intakes of fats, refined carbohydrates, and ultra-processed foods, together with low fiber consumption [4]. These eating habits not only promote excessive energy intake and weight gain but can also precipitate gastrointestinal symptoms [61]. Excessive consumption of fried or spicy foods has been associated with dyspeptic symptoms and may exacerbate IBS in susceptible individuals [62]. High-fat diets slow gastric emptying and alter gut hormone release, contributing to reflux and sensations of fullness [51]. Nutrient-poor diets rich in sugars and saturated fats promote chronic low-grade inflammation and disrupt the gut ecosystem [15,17,63,64], while insufficient dietary fiber favors constipation by producing harder, slower-transit stools [65]. Conversely, fermentable carbohydrates such as FODMAPs can elicit gastrointestinal discomfort in children with IBS, whereas restriction of these components often leads to significant symptom improvement [66].

Alterations in the intestinal microbiota further mediate the obesity–FGID connection. The composition and diversity of gut microorganisms are influenced by both dietary intake and the host’s obesity status [67,68]. Children with obesity frequently exhibit dysbiosis, marked by reduced microbial diversity and a higher Firmicutes-to-Bacteroidetes ratio compared with normal-weight peers [69]. These changes affect fermentation, gas production, and motility [70,71]. For example, in children with obesity and functional constipation, a reduction in Bacteroidetes (particularly *Prevotella* species) and an overrepresentation of certain Firmicutes have been reported [72]. Such shifts reduce the production of beneficial short-chain fatty acids while increasing methane levels, thereby slowing intestinal transit and contributing to constipation and bloating [73,74]. Dysbiosis may also compromise the intestinal barrier; indeed, obesity-associated systemic inflammation combined with microbial imbalance increases intestinal permeability (“leaky gut”) and visceral hypersensitivity [75,76], both of which may underlie recurrent abdominal pain and IBS-like symptoms.

Mechanical factors also play a role in this association. Excess intra-abdominal adiposity can increase intragastric pressure and the likelihood of hiatal hernia, impair gastric emptying, and promote gastroesophageal reflux, leading to dyspeptic manifestations [13]. In children with overweight, increased visceral fat and gastric distension may reduce gastric wall tone and alter stretch receptor sensitivity, resulting in early satiety or epigastric discomfort [13].

Hormonal and inflammatory mechanisms are likewise implicated [14]. Low-grade systemic inflammation, characterized by elevated cytokines such as interleukin-6, has been observed in adolescents with obesity and correlated with abdominal pain [14]. Obesity-related gut microbiota alterations further amplify this inflammatory state [15,16,17].

Psychological and emotional factors represent another key link between obesity and FGIDs. Children and adolescents with obesity often experience stigma, anxiety, or depression, and these psychosocial stressors are known to exacerbate gastrointestinal symptoms through the brain–gut axis [44,47,48,77,78]. Stress-related neuroendocrine activation can alter motility and visceral sensitivity, while chronic anxiety and low self-esteem may perpetuate symptom perception and eating dysregulation.

Finally, sedentary behavior contributes to both obesity and gastrointestinal dysfunction. Physical inactivity reduces intestinal motility and predisposes to constipation, whereas regular exercise has been shown to improve bowel function and overall gut health [43,79,80]. In a large prospective birth-cohort study from Rotterdam, Driessen et al. found that preschoolers in the highest tertile of physical activity at two years of age had a significantly lower prevalence of functional constipation by age four [81]. Conversely, prolonged screen time and sedentary habits are associated with irregular meal patterns and increased constipation risk [82]. Inadequate sleep hygiene—now recognized as a determinant of both pediatric obesity and FGIDs, may further disrupt appetite regulation, stress reactivity, and gastrointestinal functioning. Insufficient or poor-quality sleep alters circadian rhythms and affects key hormonal pathways, including leptin and ghrelin signaling, which can promote increased appetite, preference for energy-dense foods, and irregular eating patterns [83,84,85]. Sleep deprivation also heightens cortisol levels and autonomic arousal, potentially amplifying visceral sensitivity and lowering the threshold for gastrointestinal symptom perception. Moreover, sedentariness and excessive digital device use are linked to poorer mood and higher stress levels, which can further affect gastrointestinal function [86,87]. High screen exposure, particularly before bedtime, interferes with melatonin secretion and sleep onset, thereby compounding sleep disturbances. In addition, prolonged screen time is associated with reduced physical activity, dysregulated meal timing, such as snacking during device use, and increased risk of constipation [77,88].

### 3.3. Relationship Between UPF and FGIDs

The UPFs are characterized by high palatability, energy density, and convenience, but poor nutritional quality, being rich in free sugars, saturated fats, sodium, and additives while low in fiber, protein, and micronutrients [30,89].

Their widespread availability and targeted marketing to children have led to early-life exposure and high consumption rates, with evidence indicating that UPFs contribute up to 60% of total daily energy intake during adolescence in several cohorts [90].

Epidemiological data consistently link UPF consumption with obesity, central adiposity, and metabolic syndrome in children and adolescents across diverse populations. Literature demonstrates that diets rich in UPFs are associated with higher body mass index (BMI), waist circumference, and fat mass, independent of total caloric intake [4,27,89]. A 2024 systematic review by Petridi et al. [3] found that 14 out of 17 studies reported a positive association between UPF intake and overweight or obesity in children and adolescents. Frequent consumption of UPFs has also been correlated with increased waist circumference, elevated LDL-cholesterol and triglycerides, and insulin resistance, even at early ages [2]. Similarly, Mescoloto et al. [4] reported that children worldwide now derive a significant proportion of their total caloric intake from UPFs, with such dietary patterns consistently associated with excess weight, reduced physical activity, and heightened cardiometabolic risk. A meta-analysis also reported a dose–response relationship, with each 10% increase in energy derived from UPFs significantly increasing the risk of obesity and metabolic disorders in youth [91]. Evidence from the ERICA study further showed that Brazilian adolescents with high UPF intake displayed adverse cardiometabolic profiles, including dyslipidemia, elevated blood pressure, and insulin resistance [31]. Similar trends were observed in UK cohorts, where UPF consumption steadily increased between 2008 and 2019, particularly among lower socioeconomic groups, and correlated with adverse metabolic trajectories [90].

The introduction of UPFs during infancy is especially concerning, as early exposure predicts long-term dietary preferences and metabolic risk [92]. Children as young as 24 months consume an average of five UPF products per day, with higher intake associated with lower maternal education and household income [92]. Maternal diet itself exerts significant intergenerational effects: prospective studies show that higher maternal UPF intake during child-rearing years is associated with an increased risk of overweight and obesity in offspring, independent of the child’s own diet and lifestyle, suggesting epigenetic and microbiome-mediated mechanisms [93].

This aligns with the developmental origins of health and disease (DOHaD) framework, which highlights how maternal nutritional patterns during pregnancy and lactation can shape offspring metabolic and gastrointestinal health. High maternal UPF intake has been associated with altered placental nutrient transport, increased oxidative stress, and modifications in DNA methylation of genes regulating adipogenesis and insulin sensitivity. Moreover, maternal consumption of low-fiber, high-additive foods can perturb maternal gut microbiota, influencing microbial colonization in the neonate and predisposing to dysbiosis and inflammation early in life [93].

Table 2 summarizes the key studies that examine the association between UPF intake and FGIDS.

Mechanistic evidence indicates that UPFs can adversely affect both metabolic and gastrointestinal function through several interconnected pathways involving gut dysbiosis, impaired mucosal barrier integrity, and low-grade inflammation, mechanisms that are also implicated in the pathophysiology of functional gastrointestinal disorders in adolescence [96,97].

Diets dominated by UPFs disrupt gut microbiota composition, leading to reduced microbial diversity and decreased production of short-chain fatty acids, which play crucial roles in intestinal barrier integrity and metabolic homeostasis [98]. In pediatric cohorts, UPF-rich diets have been linked to reductions in beneficial taxa such as *Bifidobacterium*, *Faecalibacterium prausnitzii*, and *Roseburia* spp., paralleled by an overrepresentation of pro-inflammatory or opportunistic genera including *Enterobacteriaceae* and *Ruminococcus gnavus*. These microbial shifts lead to decreased production of key metabolites such as butyrate and propionate, which are essential for epithelial energy supply and anti-inflammatory signaling. The resulting metabolic imbalance promotes intestinal permeability and immune activation, reinforcing the link between UPF-driven dysbiosis and functional gastrointestinal disorders in youth [98,99].

Dysbiosis is associated with increased intestinal permeability (“leaky gut”), bacterial translocation, and low-grade systemic inflammation, all of which contribute to obesity, insulin resistance, and gastrointestinal discomfort [99]. Additionally, inadequate nutrient profiles associated with high UPF intake -particularly reduced fiber and micronutrients- may further compromise intestinal homeostasis by weakening epithelial defense and favoring dysbiotic states that predispose to gastrointestinal symptoms [96,97].

Consistent with these observations, recent reviews indicate that UPF-rich diets are associated with a higher prevalence of abdominal pain, bloating, and altered bowel habits in children and adolescents, suggesting an early contribution to the development of FGIDs [30,95].

The high glycemic load and lipid content of UPFs induce postprandial oxidative stress and metabolic endotoxemia, amplifying inflammatory responses and altering neuroendocrine regulation [97]. Processing by-products, including advanced glycation end-products, and contaminants from packaging, such as bisphenols, may further impair mucosal defense and influence neuroimmune signaling [98]. These mechanisms collectively contribute to metabolic dysfunction and gastrointestinal pathology observed in pediatric UPF consumers.

Emerging evidence also implicates UPFs in neuropsychological outcomes. Adolescents with high UPF consumption exhibit higher levels of depressive symptoms, internalizing and externalizing behaviors, and reduced psychosocial functioning [98]. These findings align with preclinical evidence showing that UPF components can impair brain regions involved in emotional regulation, including the hippocampus and cortex. The gut–brain axis likely mediates these effects: microbiota alterations, increased intestinal permeability, and systemic inflammation may influence neurotransmitter production and neuroimmune signaling, thereby contributing to mood and behavioral disorders [89,94]. Moreover, García-Blanco L. et al. [100] have highlighted that gut-related symptoms frequently accompany these neurobehavioral alterations, reinforcing the bidirectional interplay between gastrointestinal and psychological domains in UPF consumers.

Additionally, food selectivity and sensory preferences for hyper-palatable foods, often observed in children with autism spectrum disorder, further exacerbate nutritional imbalances and gastrointestinal symptoms [27].

The adverse effects of UPFs are not limited to individual health outcomes but extend to broader public health implications. The increasing prevalence of obesity, metabolic syndrome, and FGIDs in pediatric populations is likely driven, at least in part, by the growing dominance of UPFs in modern diets [89]. This trend is compounded by socioeconomic disparities, with disadvantaged populations experiencing higher UPF exposure and greater vulnerability to associated health risks [101].

Reducing UPF consumption from early life, supported by public health policies promoting healthier food environments, nutritional education, and maternal dietary interventions, represents a key strategy to mitigate the burden of metabolic and gastrointestinal disorders and improve long-term health trajectories.

**Figure 1:**
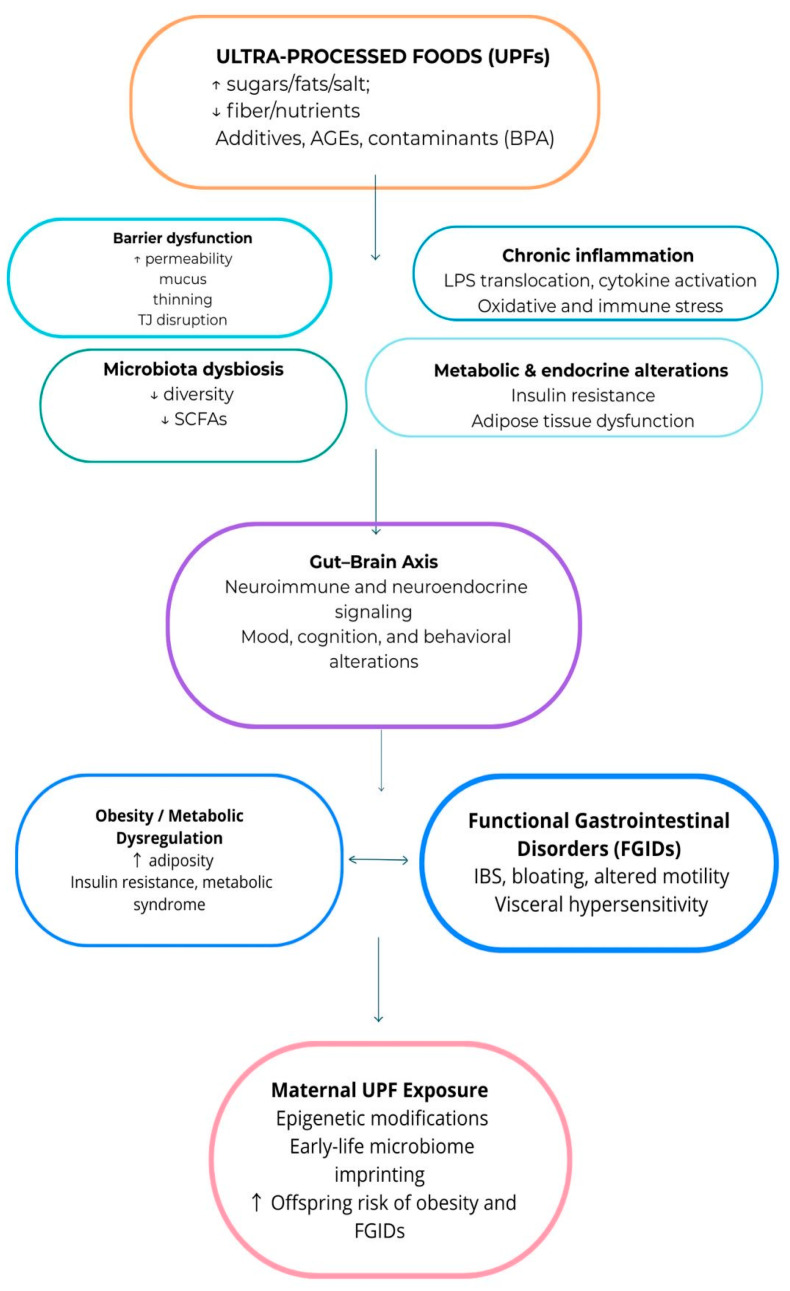
Schematic representation of the mechanisms linking ultra-processed foods (UPFs) to gut and metabolic health. UPFs, rich in sugars, fats, and additives but poor in fiber and nutrients, induce microbiota dysbiosis, barrier dysfunction, chronic inflammation, and metabolic alterations. These changes affect the gut–brain axis, leading to mood, cognitive, and behavioral disturbances, as well as obesity, metabolic dysregulation, and functional gastrointestinal disorders (FGIDs). Maternal UPF exposure may further increase offspring susceptibility through epigenetic and microbiome-mediated effects. ↑—increased; ↓—decreased.

Representation of the mechanisms linking UPFs to gut and metabolic health are schematized in Figure 1.

## 4. Prevention and Management Strategies

Preventing obesity and FGIDs in childhood requires an integrated approach that addresses diet, lifestyle, and psychosocial factors from the earliest stages of life [102]. Educational and community-based interventions promoting balanced, minimally processed diets, together with regular physical activity and emotional well-being, are fundamental to shaping healthy, lasting habits [102]. These actions are particularly relevant during childhood and adolescence, when eating patterns and reward-related behaviors are still developing.

In the decision-making process regarding food choices, the role of the reward system is particularly important. This neurobiological network is stimulated by the intake of highly palatable foods, such as UPFs and is influenced by cognitive and emotional processes that determine the perceived reward value of food. These mechanisms modulate appetite and drive eating behaviors [99]. Over time, repeated exposure to UPFs strengthens cravings for these products and reinforces habitual consumption, particularly under stress or negative emotional states. This can lead to unhealthy dietary choices and weight gain [103]. Examples of UPFs include soft drinks, flavored dairy drinks, packaged snacks, ice cream, and ready meals. These foods are easily found in vending machines at schools, where children and adolescents spend a large part of their day. Parents also play a direct role in feeding their infants and children. They provide foods to the table, establishing practical guidance on what is eaten. At the same time, they serve as meaningful role models, teaching children what, how, and when to eat. Since growing children do not have full autonomy over food choices, parental habits strongly influence children’s dietary patterns. Children are more likely to overconsume UPFs if their parents tend to do so. This emphasizes the importance of family-centered interventions to prevent and treat childhood obesity, including nutritional education and environmental interventions aimed at reducing exposure to UPFs and increasing awareness of healthier food options [40].

Socioeconomic status is another important factor influencing children’s and adolescents’ dietary patterns. In Mediterranean countries, UPFs consumption is generally lower than in other countries [104], likely due to the persistence of traditional dietary patterns, such as the Mediterranean diet, which favors fresh and minimally processed foods. However, the rapid rise in UPFs consumption and “Western” eating habits is gradually leading to the abandonment of Mediterranean dietary traditions, particularly among younger populations. Traditionally, the Mediterranean diet is based on minimally processed foods such as whole grains, legumes, fruits, vegetables, seeds, and nuts. It includes moderate consumption of fish, poultry, dairy products, and eggs, with rare intake of red meat. Simple sweets such as fruit and honey are consumed, along with olive oil as the main condiment. This dietary pattern is effective for reducing the incidence of non-communicable diseases (NCDs) and cardiovascular diseases (CVDs), in contrast to the heavy consumption of UPFs.

Interventions such as the DELICIOUS project [105] aim to increase awareness, knowledge and adherence to the Mediterranean diet among school-age children and adolescents, while reducing their consumption of UPFs. Nutritional and sustainability content based on the Mediterranean dietary model has been adapted for primary school curricula (ages 6–12), in accordance with national education policies and taking into account different ethnic groups. Additionally, new educational materials based on the Mediterranean diet have been created for children and adolescents (aged 6–17), as well as for their families, guardians, and school staff and teachers, to promote long-term adherence to the Mediterranean diet. Reducing UPFs consumption can also be indirectly aided by small-scale, low-cost initiatives that leverage social capital and local resources. These initiatives aim to transform local rural food environments and eating habits by carrying out collaborative, educational and practical activities in the local area. One example is school gardens, which can help children and adolescents experience and learn about the plant world despite the prevalence of UPFs [106]. Regardless of the relationship between UPFs energy intake and compliance with nutritional recommendations, there is a consensus that individuals adhering more closely to the Mediterranean diet tend to consume fewer UPFs [104]. Even in Mediterranean countries, individuals who consume a higher proportion of UPFs are generally younger, smokers, moderately physically active, and frequently eat out. This reflects a modern and active lifestyle and the current issue of the increasing consumption of UPFs at the expense of unprocessed or minimally processed foods, even within our European population, particularly in Northern Europe [107].

However, the translation of the Mediterranean dietary model into non-Mediterranean or low-resource settings presents several challenges. Cultural food preferences, limited access to fresh and minimally processed products, urbanization, and the higher cost of fruits, vegetables, and fish often hinder adherence to Mediterranean-like diets. In addition, aggressive UPF marketing and time constraints related to modern work schedules reduce feasibility. Nevertheless, several facilitators may promote gradual adoption, including adapting local diets to emphasize plant-based and minimally processed foods, integrating regionally available legumes, grains, and oils, and implementing community-based and school nutrition programs. Policy actions—such as subsidies for fresh produce, taxation of UPFs, and improved food labeling—may further support sustainable dietary shifts [107].

To improve public health policies and raise awareness of the risks associated with excessive UPFs consumption, several factors must be considered. These should include the growing prevalence of stressful, work-based lifestyles, the increased availability of ready-to-eat UPFs, and the limited opportunities for communal meals. Awareness campaigns could target parents and caregivers who make food choices for growing children, emphasizing the importance of fostering healthy eating habits from an early age. In fact, adopting a healthy lifestyle well before conception is vital. Studies show that the first 1000 days of life are crucial, as childhood obesity is a strong predictor of adult obesity, which carries significant health and economic consequences for individuals and society [108].

In recent years, some strategies have proven to be effective in indirectly encouraging the purchase of healthier products, such as nudging, for example, by placing healthier foods at eye level to influence choices indirectly [109].

Education and awareness-raising initiatives regarding the consumption of UPFs should be carried out at multiple levels. At the educational level, it is desirable to introduce healthier foods, such as fresh fruits and minimally processed options, into school and gym cafeterias, vending machines, and groceries. This strategy would replace foods that increase the risk of obesity and associated comorbidities with healthier alternatives [110]. Within the family framework, a multidisciplinary approach involving families and school-based educational programs can raise awareness and encourage healthier, more sustainable eating patterns, thereby reducing UPF consumption [111]. These initiatives should also consider ethnic variability and cultural differences in eating habits and affordability [112].

Although this review focuses on young people and young adults, it is evident that UPFs consumption influences dietary habits in adulthood [113]. Younger individuals consume more UPFs, often alongside unprocessed foods, while older adults generally consume fewer UPFs. However, older adults with higher UPF intake also tend to consume fewer unprocessed foods and have a less varied diet [114].

In light of all of this information, considering the strong associations between UPFs consumption and obesity and other health issues, monitoring UPFs consumption across all age groups is essential. This is especially important in populations whose diets diverge significantly from the Mediterranean diet and whose traditional lifestyles involve lower UPFs consumption, as it can help predict potential increases in NCDs [113].

## 5. Future Perspectives

The relationship between pediatric obesity, UPFs consumption, and FGIDs is complex and multifactorial. Future research should focus on high-quality pediatric trials to define effective preventive and therapeutic interventions, as current evidence largely derives from adult studies. Randomized controlled trials are needed to assess dietary approaches, such as low-FODMAP diets, fiber supplementation, and microbiome-modulating therapies (probiotics, prebiotics, synbiotics), in children with obesity and FGIDs, using standardized outcomes and adequate follow-up to evaluate long-term efficacy.

Clarifying the role of the gut microbiota in mediating the link between UPFs, obesity, and gastrointestinal symptoms is another research priority. Excessive UPF intake alters microbial diversity, intestinal permeability, and inflammatory responses, but pediatric data remain scarce. Understanding how prolonged UPF exposure affects the developing gut microbiota may help identify microbial biomarkers and novel therapeutic or dietary targets.

Future work should adopt interdisciplinary and multi-omics approaches combining gastroenterology, nutrition, and microbiome science to elucidate the mechanisms connecting diet, microbes, and host physiology. Personalized interventions based on each child’s microbiota, genetics, and behavioral profile are also essential, as both obesity and FGIDs are heterogeneous and influenced by psychosocial factors. Precision nutrition and microbiome-guided dietary strategies, supported by adult evidence, may improve gastrointestinal and metabolic outcomes.

Finally, integrating psychosocial and behavioral dimensions, such as stress, sedentary habits, and emotional eating, into prevention programs could enhance their sustainability. Coordinated efforts among clinicians, researchers, and policymakers are crucial to bridge current knowledge gaps and shift clinical practice toward targeted, evidence-based, and personalized strategies capable of breaking the obesity, FGID cycle early in life and promoting better long-term health.

## 6. Limitations of the Reviewed Studies

Despite the growing body of literature addressing UPF consumption, pediatric obesity, and FGIDs, several limitations should be acknowledged.

First, most available studies are observational, limiting the ability to infer causal relationships. Self-reported dietary assessments, frequently used in UPF research, are prone to recall bias and misclassification, especially in children. Additionally, inconsistencies across UPF classification systems, such as NOVA, EPIC, and SIGA—complicate comparisons between studies and may influence outcome interpretation.

Pediatric data on microbiota alterations associated with UPF intake remain limited, and most findings have been extrapolated from adult populations. Heterogeneity in study populations, diagnostic criteria for FGIDs, and outcome measures also contributes to variability in reported results. Moreover, few studies account for important confounding factors such as physical activity, sleep patterns, socioeconomic status, and psychosocial stressors, all of which may shape dietary habits and gastrointestinal symptoms.

These limitations highlight the need for standardized methodologies, validated dietary assessment tools, and well-designed pediatric trials to strengthen the evidence base.

## 7. Conclusions

The evidence reviewed indicates a consistent association between UPF consumption, pediatric obesity, and FGIDs, suggesting that dietary patterns characterized by high intakes of ultra-processed foods may contribute not only to excessive weight gain but also to gastrointestinal dysfunction through metabolic, microbial, inflammatory, and neuroendocrine pathways. These shared mechanisms help explain the frequent coexistence of obesity and FGIDs in children and adolescents.

The rising prevalence of UPF consumption from early life, combined with its displacement of nutrient-dense foods rich in fiber, antioxidants, and anti-inflammatory compounds, underscores the need for preventive efforts centered on fresh and minimally processed foods. Dietary patterns such as the Mediterranean diet remain key strategies for preserving metabolic and gastrointestinal health during growth.

From a clinical perspective, pediatricians and nutrition specialists should consider UPF intake as a relevant factor when evaluating children with obesity and gastrointestinal complaints. A multidisciplinary approach that integrates nutritional guidance, behavioral and psychosocial support, and lifestyle modification is essential for preventing the perpetuation of the obesity–FGID cycle.

It is also important to emphasize that not all UPFs are nutritionally equivalent and that health risks depend not only on processing level but also on ingredient quality, additive content, and dietary context. The absence of a universally validated classification system further complicates interpretation across studies, reinforcing the need for a nuanced, evidence-based approach that avoids oversimplified messages.

Ultimately, investing in education, research, and public health initiatives aimed at reducing UPF consumption and promoting healthier food environments represents a cornerstone for safeguarding the metabolic and gastrointestinal health of future generations.

Investing in education, research, and public health measures aimed at reducing UPF consumption represents a cornerstone for safeguarding the metabolic and gastrointestinal health of future generations.

## Figures and Tables

**Table 1:** Main studies investigating the relationship between obesity and functional gastrointestinal disorders (FGIDs).

Author (Year)	Study Design/Population	Main Findings	Notes/Mechanistic Insights
Teitelbaum et al. (2020) [54]	Cross-sectional, pediatric gastroenterology population n = 757 patients + 255 matched controls	23% of children with functional constipation (FC) and 24.8% with IBS were obese—significantly higher than controls.	Indicates strong association between obesity and FGIDs, particularly FC and IBS.
Galai et al. (2020) [10]	Cross-sectional, adolescents n = 173 patients	39.5% of adolescents with functional abdominal pain (FAP) were overweight or obese, compared to 30% of controls.	Suggests excess weight may predispose to abdominal pain syndromes.
Tambucci et al. (2019) [11]	Observational, children/adolescents n = 103 patients + 115 matched controls	FGIDs were more common in youths with obesity (47.6%) than in normal-weight peers (17.4%). FC, FD, and IBS more frequent in obese group.	Highlights differential prevalence of FGID subtypes by weight status.
Phatak et al. (2014) [57]	Cross-sectional, school-age children n = 450 children (191 with obesity/overweight and 259 normal weight)	Overall prevalence of FGIDs: 16.1% in overweight/obese vs. 6.9% in normal-weight children.	Nearly half of obese children had ≥1 FGID, showing frequent coexistence of these conditions.
Zia JK et al. (2023) [58]	Systematic review n = 348 studies	Confirmed consistent link between obesity and FGIDs, particularly constipation; less consistent for IBS.	Suggests overlapping pathophysiological mechanisms involving gut motility and diet.
Eslick et al. (2012) [59]	Meta-analysis, adults n = 21 studies	Obesity associated with multiple GI symptoms, especially upper-abdominal symptoms (reflux, bloating).	Indicates similar trends across age groups; shared metabolic and mechanical mechanisms.
Bonilla et al. (2011) [55]	Prospective cohort study n = 188 patients	Obesity is associated with poor outcome and disability at long-term follow-up in children with abdominal pain-related FGIDs.	Highlights a strong association between persistence of GI symptoms at long-term follow-up and obesity and evidences that obese patients experienced higher intensity and frequency of FAP in patients without obesity.

Legend: FGID = Functional Gastrointestinal Disorder; IBS = Irritable Bowel Syndrome; FC = Functional Constipation; FD = Functional Dyspepsia; FAP = Functional Abdominal Pain; GI = Gastrointestinal.

**Table 2:** Main studies investigating the relationship between ultra-processed food (UPF) consumption and functional gastrointestinal disorders (FGIDs).

Author (Year)	Study Design/Population	Main Findings	Mechanistic Insights/Notes
Cuevas-Sierra et al. (2019) [21]	Cross-sectional, adults (Spain, n = 186)	Higher UPF intake (>5 servings/day) associated with altered gut microbiota composition.	Reduced microbial diversity; sex-related differences; potential link with GI symptoms.
Mescoloto et al. (2024) [4]	Review, global pediatric data	UPFs provide 40–60% of daily energy in youth; correlated with obesity and GI discomfort.	Diets low in fiber and high in additives predispose to dysbiosis and inflammation.
Petridi et al. (2024) [3]	Systematic review (17 studies, children/adolescents)	14 studies reported positive association between UPFs and overweight/obesity; FGIDs frequently co-occur.	Suggests common pathways via microbiota disruption and low-grade inflammation.
Reales-Moreno M. et al. (2022) [94]	Observational study; adolescents (n = 560)	High UPF consumption correlates with greater GI symptom burden and psychosocial impairment.	Suggests involvement of the gut–brain axis in diet-related FGID manifestations.
Belli D.C. & Gupta S.K. (2022) [95]	Narrative review summarizing key pediatric gastroenterology findings of 2021	Highlights growing concern regarding UPF exposure in children due to its association with GI disturbances, obesity, and metabolic dysregulation.	Reinforces clinical relevance of diet–gut interactions, supporting UPFs as contributors to functional GI symptoms and broader digestive health burden in pediatrics.
García-Blanco L. et al. (2023) [96]	Prospective cohort study; 806 Spanish children (SENDO project)	High UPF consumption was associated with an increased risk of micronutrient inadequacy and poorer overall diet quality.	Nutrient deficiencies linked to UPF intake may impair gut barrier integrity and microbiota composition, favoring inflammation and functional GI disturbances.
Souza S.F. et al. (2022) [97]	Observational study; adolescents (n = 576)	UPF-rich diets correlate with abdominal obesity and GI symptoms.	Shared mechanisms include inflammation and microbiota dysbiosis.
Chen X. et al. (2020) [30]	Systematic review; general population (~334,114 participants total across studies)	UPF intake is consistently associated with GI complaints and functional symptoms.	Reduced microbial diversity, chronic inflammation, and increased gut permeability are key mechanisms.
De Amicis R. et al. (2022) [40]	Observational studies; children and adolescents	UPF intake is associated with GI dysfunction and metabolic alterations.	Combined effects on gut microbiota, barrier function, and systemic inflammation likely underlie FGIDs.

UPF = Ultra-Processed Food; FGID = Functional Gastrointestinal Disorder; GI = Gastrointestinal.

## Data Availability

No new data were created or analyzed in this study. Data sharing is not applicable to this article.
